# Anesthetic Alterations of Collective Terahertz Oscillations in Tubulin Correlate with Clinical Potency: Implications for Anesthetic Action and Post-Operative Cognitive Dysfunction

**DOI:** 10.1038/s41598-017-09992-7

**Published:** 2017-08-29

**Authors:** Travis J. A. Craddock, Philip Kurian, Jordane Preto, Kamlesh Sahu, Stuart R. Hameroff, Mariusz Klobukowski, Jack A. Tuszynski

**Affiliations:** 1 Departments of Psychology & Neuroscience, Computer Science, and Clinical Immunology, and the Clinical Systems Biology Group, Institute for Neuro-Immune Medicine, Nova Southeastern University, Fort Lauderdale, Florida USA; 2 National Human Genome Center and Department of Medicine, Howard University College of Medicine, and Computational Physics Laboratory, Howard University, Washington, DC, USA; 3 Department of Experimental Oncology, Cross Cancer Institute, Edmonton, Alberta Canada; 4 Department of Physics, University of Alberta, Edmonton, Alberta Canada; 5 Department of Medical Microbiology and Immunology, University of Alberta, Edmonton, Canada; 6 Departments of Anesthesiology and Psychology, Center for Consciousness Studies, The University of Arizona Health Sciences Center, Tucson, Arizona USA; 7 Department of Chemistry, University of Alberta, Edmonton, Alberta Canada

## Abstract

Anesthesia blocks consciousness and memory while sparing non-conscious brain activities. While the exact mechanisms of anesthetic action are unknown, the Meyer-Overton correlation provides a link between anesthetic potency and solubility in a lipid-like, non-polar medium. Anesthetic action is also related to an anesthetic’s hydrophobicity, permanent dipole, and polarizability, and is accepted to occur in lipid-like, non-polar regions within brain proteins. Generally the protein target for anesthetics is assumed to be neuronal membrane receptors and ion channels, however new evidence points to critical effects on intra-neuronal microtubules, a target of interest due to their potential role in post-operative cognitive dysfunction (POCD). Here we use binding site predictions on tubulin, the protein subunit of microtubules, with molecular docking simulations, quantum chemistry calculations, and theoretical modeling of collective dipole interactions in tubulin to investigate the effect of a group of gases including anesthetics, non-anesthetics, and anesthetic/convulsants on tubulin dynamics. We found that these gases alter collective terahertz dipole oscillations in a manner that is correlated with their anesthetic potency. Understanding anesthetic action may help reveal brain mechanisms underlying consciousness, and minimize POCD in the choice and development of anesthetics used during surgeries for patients suffering from neurodegenerative conditions with compromised cytoskeletal microtubules.

## Introduction

Anesthesia is one of the world’s greatest serendipitous pharmacological discoveries, selectively and reversibly blocking consciousness while sparing non-conscious brain activities, enabling modern surgery. Yet, the mechanism by which anesthesia acts, and how the brain produces conscious experience remain unknown. Understanding anesthesia may help explain consciousness, and *vice versa*. On a more practical level, discovering sites and mechanisms of anesthetic action can help in clinical decisions (e.g. the choice of anesthetic in patients with cancer, neurodegenerative, and other disorders), lead to new anesthetics, and reduce the risk of anesthesia-related post-operative cognitive dysfunction (POCD).

The link between anesthesia, POCD, and the exacerbation of neurodegenerative disorders is a major concern^1–4^. After surgery at least 30% of adults show cognitive dysfunction two days following surgery, and lasting up to at least 3-months in 12% of the elderly^5^. This is clearly problematic for patients with neurodegenerative conditions, with anesthesia having a significant impact on disease progression and cognitive decline^6–14^. As Alzheimer’s disease (AD) and other dementias currently cost the American taxpayer $236 billion annually^15^ and with increasing anesthesia required for age-related surgery, this cost is only expected to rise as the population ages.

**Figure 1:**
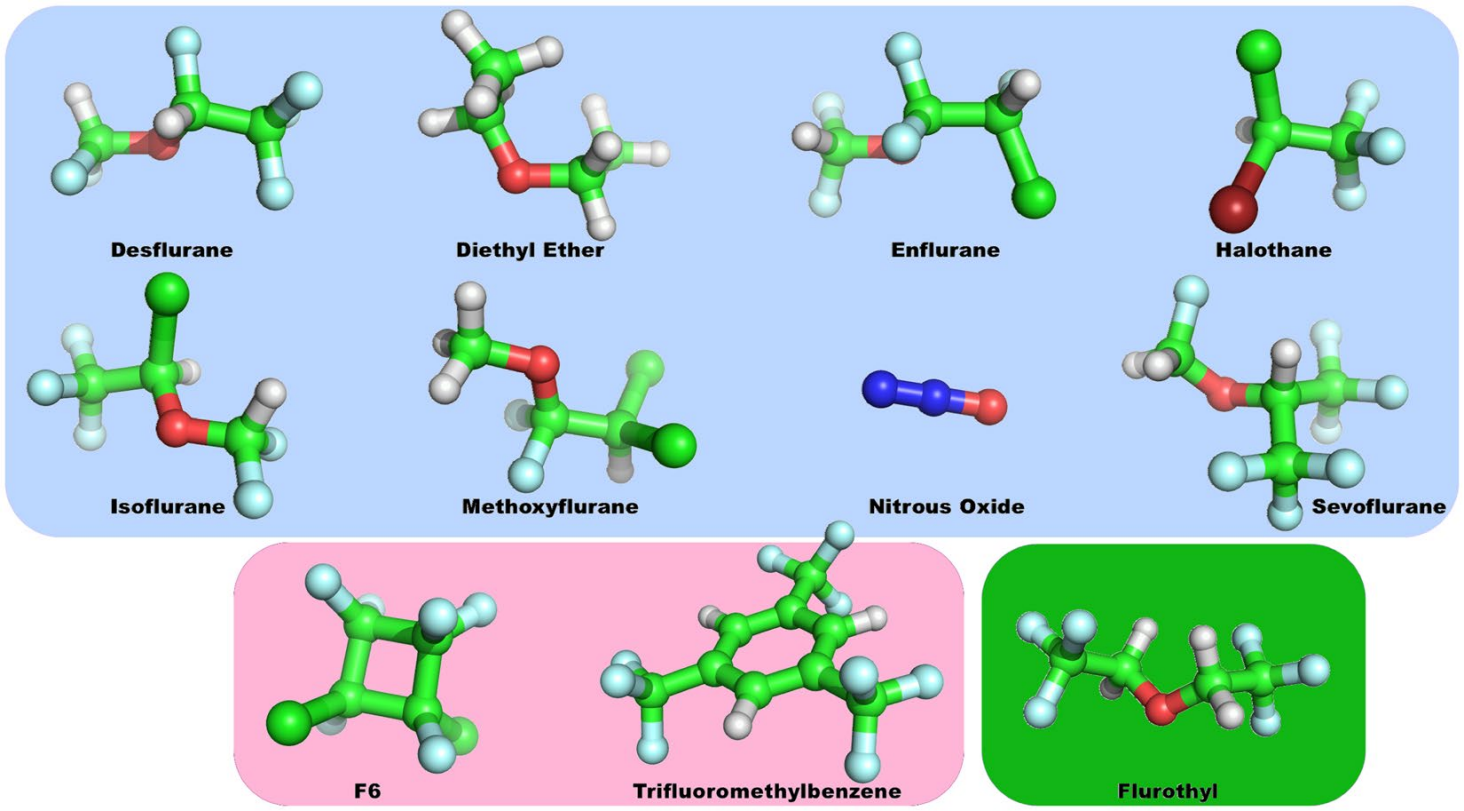
Chemical structure of investigated agents. Blue – anesthetics; Red - non-anesthetics; Green – anesthetic/convulsant.

**Figure 2:**
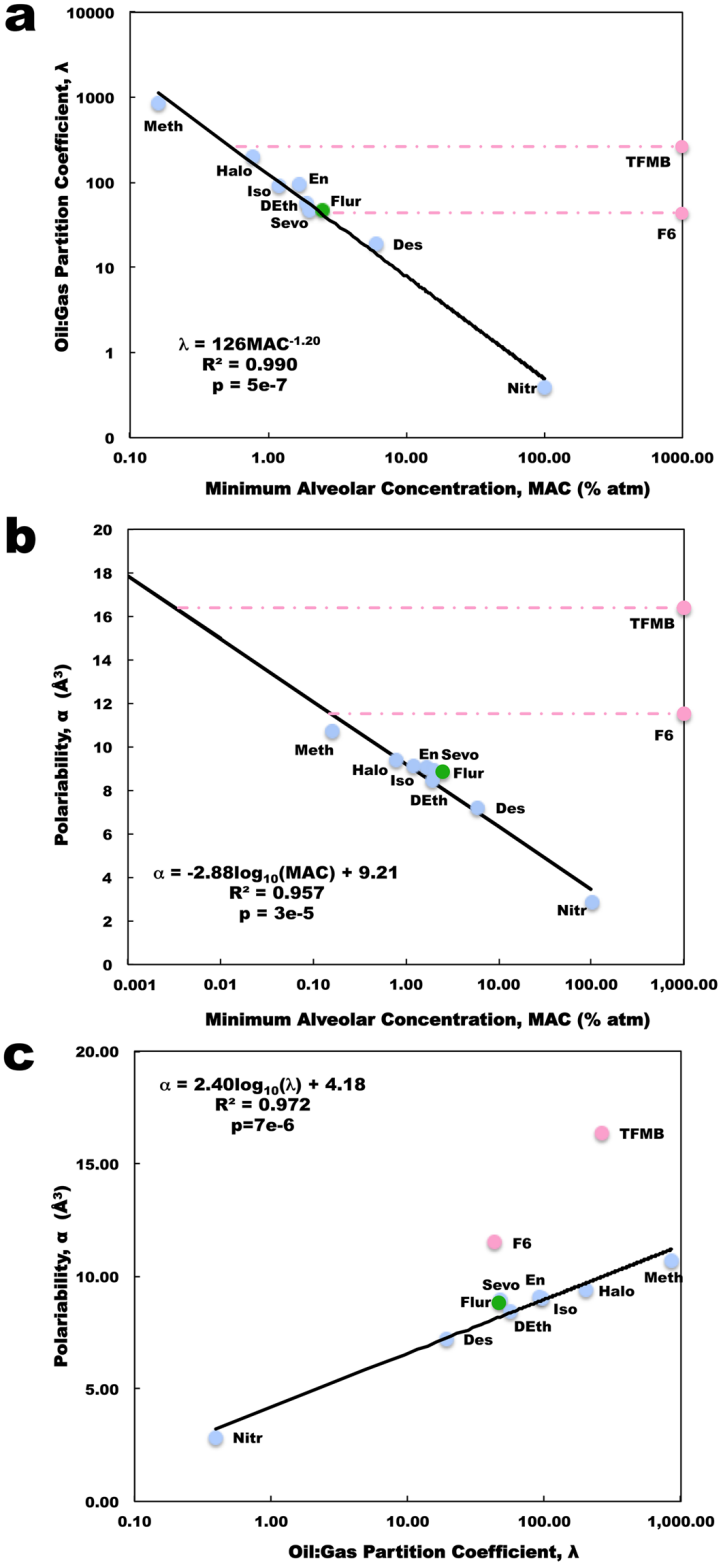
Correlation of anesthetic properties with anesthetic potency. (*a*) Meyer-Overton correlation of oil:gas partition coefficient versus MAC (Blue points – anesthetics; Red points – non-anesthetics; Green points – anesthetic/convulsant; Red line – difference between non-anesthetic predicted and estimated (~1000% atm) MAC). (*b*) Correlation of polarizability versus MAC, with MAC for non-anesthetics determined from the Meyer-Overton correlation. (*c*) Correlation of polarizability versus solubility shows a difference in the relation between these properties for non-anesthetics and anesthetics. Trend lines and equations based on anesthetics alone, without the contributions from the non-anesthetics and anesthetic/convulsant.

The mystery of anesthesia stems from the baffling structure–activity relationship of general anesthetics, as effective agents can span a 35-fold range in molecular volume from a single atom (xenon) to 56-atom steroids, with numerous types of chemical structures, including ethers and halogenated hydrocarbons, in between^16^ (Fig. 1). Examining the anesthetic action of gases with such disparate structures, Hans Meyer^17^ and Charles Overton^18^ discovered that anesthetic potency (e.g. the inverse of the minimum alveolar concentration (MAC) at which half of animals tested would lose purposeful behavior) correlates highly with their solubility in a particular non-polar solvent akin to olive oil^17, 18^. Thus, the ‘Meyer-Overton correlation’ revealed that the anesthetic potency of a gas molecule correlates with its solubility in a non-polar, ‘lipid-like’, hydrophobic (i.e. water excluding) medium (Fig. 2A, Table S1). Surprisingly, this correlation holds for general anesthetic activity in many organisms^16^ from paramecia to humans^19^, and even plants^20–22^. Furthermore, anesthesia is completely reversible.

The Meyer-Overton correlation suggests a common, unitary mechanism of anesthetic action. Initially this was taken to imply critical effects on the excitability of lipid bilayers in neuronal membranes. However, anesthetic effects on lipids cannot account for differences between mirror image ‘chiral’ anesthetic molecules^23^, the ‘cut-off’ effect (lack of anesthetic effect of molecules which follow Meyer-Overton but are too large, e.g. increasing length of n-alcohols^24^ or n-alkanes^25^), or the lack of anesthesia by temperature induced re-ordering in lipids which mimics anesthetic effects therein^26^. Finally, there are exceptions to the Meyer-Overton rule. Agents, such as 1,3,5 tris(trifluoromethyl)benzene (TFMB) and 1,2 dichlorohexafluorocyclobutane (F6), which are predicted by their lipid solubility to have significant anesthetic potency but do not, even at higher concentrations (represented by an estimated MAC of 1000% atmospheres (atm)) (Fig. 2A). Consequently, these agents are called non-anesthetics. Other gases, such as flurothyl (indoklan), show unresponsiveness at their Meyer-Overton predicted anesthetic potentcy, but cause seizures at lower concentrations and are thus denoted as anesthetic/convulsants^27^. Discerning how non-anesthetics differ from anesthetics may be an important clue to understanding anesthesia.

It is well accepted that the effect of anesthetics is related to their hydrophobicity, as well as their permanent dipole strength, and polarizability^28, 29^. Considering the problems with the lipid bilayer theories of anesthesia, Franks and Lieb demonstrated that the Meyer-Overton correlation was consistent with anesthetics acting directly on proteins, specifically in non-polar, lipid-like ‘hydrophobic pockets’ within protein interiors^30^. These non-polar intra-protein regions provide for a chiral environment, and therefore, the orientation of the pocket-bound anesthetic is determined by its structure and permanent dipole moment. Furthermore, these regions are composed largely of highly polarizable, π-resonance clouds of aromatic amino acids such as tryptophan, tyrosine, and phenylalanine. Polarizability, which refers to the degree to which a molecule can be instantaneously polarized by nearby charge distributions, correlates strongly with anesthetic potency^28, 29^ (Fig. 2B, Table S1). However, the polarizability correlation also implies that the predicted MAC of the non-anesthetics F6 and TFMB are less than predicted by the Meyer-Overton relation suggesting an even higher anesthetic potency for these agents than previously believed, but this is clearly not the case as they have no anesthetic effect even at significantly higher doses. What this does indicate is that there is a clear deviation from the correlation between the physical parameters for lipid solubility and molecular polarizability for these non-anesthetic agents compared to anesthetics (Fig. 2C, Table S1). Thus, it appears that non-polar ‘lipid’ solubility determines anesthetic binding at the site of action, i.e. within hydrophobic pockets, but how does this cooperate with molecular polarizability to act in the brain and cause loss of consciousness?

Anesthetic binding within protein interiors is thought to block critical conformational changes or other specific dynamic protein functions, but the question of which proteins are critically involved remains unclear. Franks and Lieb, and many others, assumed that anesthetics target membrane receptor and ion channel proteins, as these directly govern dendritic-somatic membrane excitability and depolarization, which govern axonal firing rates. Specifically, post-synaptic GABA_A_, acetylcholine, serotonin, glutamate, glycine, α2-adrenergic, acetylcholine, or adenosine receptors have been the presumptive membrane protein targets, and anesthetics have been shown to have a high binding affinity for these sites. However, under anesthesia, there’s more anesthetic in the peripheral fat stores of the patient’s body than in their brain, yet anesthetics act in the brain. Thus, anesthetics clearly bind to these receptors and channels at their MAC value, but as anesthetic actions on them are highly variable and inconsistent, they have been deemed fruitless in terms of a common mechanism of action^31^.

Yet, while membrane- bound receptors and ion channels continue to be considered the primary sites of anesthetic action, the microtubule cytoskeleton inside neurons remains overlooked^32^. In 1968, Allison and Nunn showed that the anesthetic halothane caused depolymerization of microtubules, although at high anesthetic concentrations of about 5 times their MAC value^33^. While this is beyond clinical concentrations, a systematic approach by the Eckenhoff lab using radiolabeled halothane in mice showed that at clinically relevant concentrations (~the MAC value for mice), anesthetics bind to ~70 different neuronal proteins, half in membranes and half in the cytoplasm^34^. Among these was cytoskeletal tubulin, the component subunit protein of microtubules. Many studies since have indicated direct binding of several anesthetics directly to tubulin including halothane^34, 35^, 6-Azi-pregnanolone^36^, and 1-azidoanthracene^37^. Furthermore, proteomic analysis of genetic expression following exposure to the volatile anesthetics halothane, isoflurane, desflurane, and sevoflurane show alterations in tubulin gene expression several days after treatment^38–40^ with no changes observed in the expression of membrane proteins^38^. Clearly, anesthetics do bind to membrane receptors and channels as well as lipids at their MAC value, but they also bind to tubulin in microtubules.

These findings are of relevance because learning, memory, cognition, and the long-term potentiation paradigm specifically require a cytoskeleton capable of complex reorganization to accommodate changes in synaptic activity and strength^41–45^. This further suggests that anesthetic-induced changes in cytoskeletal stability may be a common mechanism for anesthesia^46^. Neurodegenerative diseases share the pathology of a dysfunctional neuronal cytoskeleton (e.g. Alzheimer’s, Parkinson’s etc.)^47^, and since the architecturally complex cytoskeletal matrix within neurons is responsible for neuron morphology and intracellular transport, the interaction of anesthetics with tubulin and microtubules is important for understanding the effects of anesthesia-induced post-operative cognitive dysfunction (POCD).

While this is clearly an issue, the mechanisms leading to cognitive impairment after anesthesia and surgery are not yet fully understood. It has been previously hypothesized that anesthetics can alter resonance in π-electron cloud oscillations among highly polarizable non-polar amino acids in tubulin^46^. Overall, dipoles can be induced within these electron clouds by nearby charges, dipoles, or other polarizable structures. When in proper orientation π-resonance structures (exemplified in simplest form by benzene) attract each other by van der Waals-type London dispersion forces, which then couple and oscillate. As dispersion forces tend to be stronger between molecules that are easily polarized, and as these dispersion forces contribute to protein folding and protein-protein interactions^48^, the effect of anesthetic polarizability on tubulin has implications for the dynamics of microtubule stability during and after surgery.

As any mechanism of anesthesia is expected to discriminate between true anesthetics and non-anesthetics, we aim to further test this hypothesis by assessing the effects of several volatile anesthetics, the non-anesthetics F6 and TFMB, as well as the convulsant flurothyl on the London dispersion interactions between highly polarizable aromatic amino acids in tubulin. To investigate how molecular solubility and polarizability may contribute to anesthetic action we use results of previous anesthetic binding site predictions on tubulin with molecular docking, quantum chemistry calculations, and theoretical modeling of collective London dispersion interactions to investigate the effects of this group of gases on tubulin function. As this mechanism has direct bearing on the link between anesthesia, post-operative cognitive disorder (POCD) and its effect on neurodegenerative disease, it has the potential to provide new insights on the site and mechanism of anesthetic action, and may in the future contribute to the design and development of new anesthetics with fewer potentially harmful side effects. Below, we discuss the results obtained in our study.

## Results

### Anesthetic Docking to Tubulin

To computationally predict the effect of anesthetics on the tubulin protein we ran docking simulations of 8 anesthetic molecules, 2 non-anesthetic molecules, and the convulsant flurothyl (Fig. 1), on previously predicted high affinity sites of binding to tubulin^32^. The results of anesthetic docking to tubulin are summarized in Table S2.

Successful docking was completed for all 9 high-affinity binding sites for all 11 agents except for the non-anesthetic F6 to tubulin site 5 and non-anesthetic TFMB to site 7. Most likely these agents failed to dock due to the binding site being too small or containing unfavorable functional groups, donor/acceptor interactions, hydrogen bonds or hydrophobic effects. Whether this relates to their non-anesthetic effect is unknown. All binding energy values range between −4 and −12 kJ/mol, which is indicative of weak non-covalent interactions consistent with the known binding action of anesthetic agents by van der Waals-type London dispersion forces.

To determine if there is a preferred binding site on tubulin for all anesthetics, ANOVA analysis was run to compare anesthetic binding at each site. Figure S1 provides a graphical illustration of the distribution of binding sites of the various anesthetics on tubulin. On average, the preference for anesthetics to bind at a tubulin site are ordered as site 7, 5, 38, 4, 1, 23, 37, and finally 21. However, statistical difference (p < 0.05) was only found between site 21 and sites 4, 5, 7 and 38. No other significant differences were found, suggesting that there is no overall preferred binding site for anesthetics to tubulin. Still, it is worth noting that sites 7 and 38 are within 5 angstroms of key residues of the colchicine-binding pocket which is consistent with the volatile anesthetic halothane being shown to reduce colchicine binding to tubulin^49^. Furthermore, sites 5, 7, and 38 are all within 5 angstroms of residues that interact with either the intradimer non-exchangeable GTP molecule or the magnesium ion required for the intradimer alpha-beta tubulin stability, and may be a mechanism by which volatile anesthetics disrupt microtubule structure^50^.

### Quantum Chemical Estimate of Polarizability and MAC

We calculated the mean polarizability and polarizability tensors for all 11 agents listed in Fig. 1 using a density functional theory approach. The results of our quantum chemical estimate of mean polarizability are shown in Fig. 2B and listed in Table S1, along with experimental measures of anesthetic MAC and predicted MAC for non-anesthetics and convulsants. However, these estimates are based on the mean polarizabilities of the molecules and does not account for the directions of polarization, permanent dipole moments, or docking orientation of agents to a given protein.

To investigate the full effect of anesthetic, non-anesthetic, and convulsant polarizabilities and permanent dipole moments on the collective electronic behavior of the tubulin dimer, we calculated the molecular dipole components induced by London dispersion forces between the highly polarizable aromatic amino acids tryptophan, tyrosine, and phenylalanine based on their molecular polarizability tensors, and evaluated the change between collective dipole oscillations in the presence and absence of the agent molecules. In the absence of agent molecules the aromatic amino acids of tubulin set up normal oscillatory modes that range in frequency between 480 and 700 THz (1 THz = 10^12^ Hz). This result provides a first-order description of the collective oscillation in tubulin. A full quantum mechanical parametrization of the coupled atomic dipolar fluctuations in valence electronic response, as done in the many-body dispersion approach to describe collective wavelike charge density fluctuations^51^, would provide a more refined estimate of this behavior.

**Figure 3:**
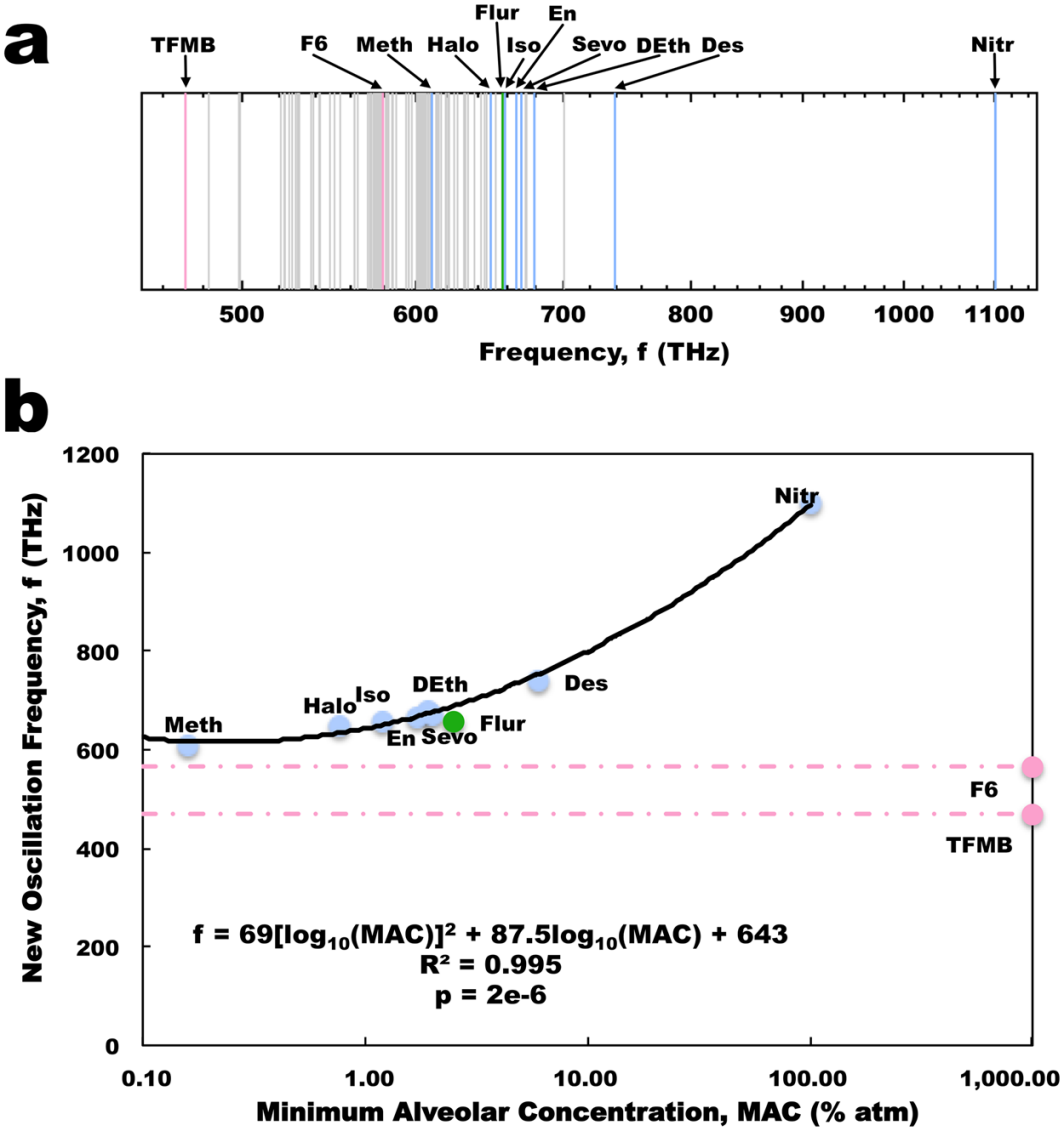
Collective dipole modes of oscillation in tubulin. (*a*) Average energies of the collective dipole modes of oscillation in tubulin. Gray – normal modes predicted for tryptophan, tyrosine and phenylalanine in tubulin in the absence of agents. (Blue – additional normal modes introduced due to the presence of an anesthetic agent; Red - additional normal modes introduced due to the presence of a non-anesthetic agent; Green – additional normal mode introduced to the presence of the anesthetic/convulsant agent flurothyl). (*b*) Agent-induced new frequency modes of oscillation versus MAC. As the non-anesthetics fall below the trend line minimum there is no predicted MAC for non-anesthetics available at any value. (Blue points – anesthetics; Red points - non-anesthetics; Green points – anesthetic/convulsant; Red line – difference between non-anesthetic predicted and actual (~1000% atm) MAC).

The presence of an agent molecule creates another normal mode of dipole oscillation for the aromatic-agent network. In Fig. 3 we plot these new normal modes created by the addition of an agent as a function of their MAC. A clear polynomial trend can be seen for anesthetics alone, with a very high degree of correlation (R^2^ = 0.995), which is slightly greater than that found for the Meyer-Overton relation (Fig. 2A). While the anesthetic/convulsant flurothyl also follows this trend, the non-anesthetics both do not. Rather, due to the polynomial nature of the relation the minimum possible frequency that would lie on the curve is 594 THz, which is marginally greater than both of the frequencies introduced by the non-anesthetics investigated, suggesting the non-anesthetics fall below a cutoff required for anesthetic action. The biological importance of this range is not fully understood at this time.

**Figure 4:**
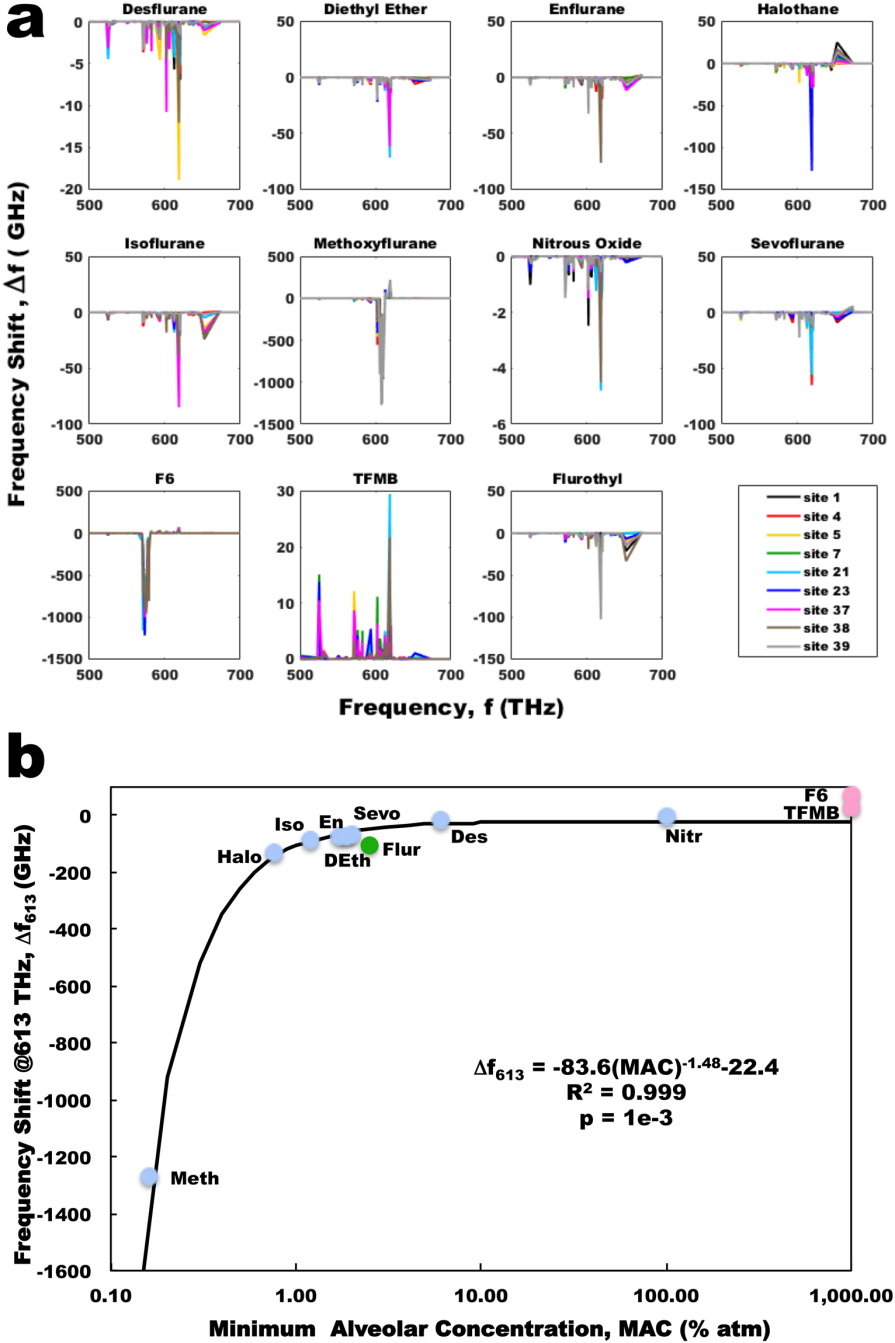
Change in tubulin collective dipole modes due to the addition of anesthetic/non-anesthetic molecules for different binding sites. (*a*) Site specific changes for anesthetics and anesthetic/convulsant flurothyl shows a prominent downwards shift at (613 ± 8) THz, while non-anesthetics F6 and TFMB show an increase at this frequency band. (*b*) Maximum agent induced change in tubulin normal mode oscillation frequency at (613 ± 8) THz versus agent MAC. (Blue points – anesthetics; Red points - non-anesthetics; Green points – anesthetic/ convulsant).

The introduction of an agent also shifts the normal oscillatory modes of the aromatic dipoles by an order of 1 to 100 GHz (1 GHz = 10^9^ Hz). Figure 4A shows specific plots illustrating the shifts in the oscillation frequencies of tubulin’s aromatic amino acids for agents docked at each of the predicted tubulin binding sites. No correlation between peak shift in frequency and binding site energy was found. The most prominent shift observed for all the anesthetic agents was observed as a decrease in the normal mode of oscillation in the range of (613 ± 8) THz. Both the biological significance of this range and the relevance of a decrease in oscillation frequency to anesthetic action, versus an increase, require further investigation. The maximum frequency decrease in this range induced by anesthetics correlates with MAC (R^2^ = 0.999) (Fig. 4B). Flurothyl again followed the same trend as the anesthetics, while F6 and TFMB did not show any decreases in this range, but rather showed increases in oscillation frequency. Of note is that the predicted MAC of the non-anesthetics is greater than 1000% atm well beyond physiologically relevant concentrations, correctly predicting their lack of anesthetic action.

## Discussion

Anesthesia is one of the great achievements of modern medicine, yet the mechanisms by which anesthetics selectively block consciousness and memory formation, while sparing non-conscious brain activities, remain unclear in spite of a century and a half of clinical use. Understanding anesthetic mechanisms may shed light on several problems in consciousness studies, which would be an important scientific and philosophical achievement. More practically, a better understanding of anesthesia can aid in clinical decisions to reduce the risk of adverse effects including POCD, particularly in those suffering from neurodegenerative conditions, and/or lead to the rational design and development of anesthetics with fewer deleterious side effects.

Yet how do anesthetic gases, a disparate group of volatile chemical compounds, exert a common, unitary effect in all animals at concentrations specific to each gas? The Meyer-Overton correlation implies a unifying factor related to the solubility of a gas in lipid-like, non-polar hydrophobic solvents. Binding in these solvents occurs by very weak London dipole dispersion, a type of weak van der Waals force, but the significance of the Meyer-Overton correlation remains elusive. Solubility in lipids alone does not account for the ‘cut-off’ effect^24, 25^, anesthetic differences due to chirality^23^, the lack of other lipid-altering effects to cause anesthesia^26^, or the role of membrane proteins in neuron excitability, leading to the conclusion that anesthetics act in non-polar, hydrophobic regions within proteins. Furthermore, there are exceptions to the Meyer-Overton rule where an agent’s lipid solubility predicts it to have a significant anesthetic potency, but it does not (i.e. F6, TFMB). These exceptions, however, may provide insight into the mechanisms of anesthetics.

Examining how molecular polarizability correlates with anesthetic potency reveals a discontinuity with the Meyer-Overton rule, specifically for the non-anesthetics F6 and TFMB. What is the difference in relation between non-polar, ‘lipid’ solubility and polarizability such that these gases don’t cause anesthesia? In this paper we investigate this relationship and present a computational study aimed at examining the effect of anesthetic, non-anesthetic, and anesthetic/convulsant agent polarizabilities and permanent dipole moments on protein function.

While membrane receptors and ion channel proteins are the assumed target of anesthetic action, genomic and proteomic studies^34–38^ point to microtubules as a functional target of anesthetic action. Relating anesthetic action to microtubules is relevant clinically because learning, memory, cognition, and the long-term potentiation paradigm specifically require a cytoskeleton capable of complex reorganization to accommodate changes in synaptic activity and strength^41–45^. Furthermore, this suggests that anesthetic effects on microtubules which may affect cytoskeleton stability may be a common mechanism for anesthesia^46^. As neurodegenerative diseases share the pathology of a dysfunctional neuronal cytoskeleton (e.g. Alzheimer’s, Parkinson’s etc.)^47^, and since the architecturally complex cytoskeletal matrix within neurons is responsible for neuron morphology and intracellular transport, the interaction of anesthetics with tubulin and microtubules may be important for understanding the effects of anesthesia-induced POCD. As dispersion forces tend to be stronger between molecules that are easily polarized, and as these dispersion forces contribute to protein folding and protein-protein interactions^48^, the effect of anesthetic polarizability on tubulin has implications for dynamics and stability of microtubules during and after surgery. As such we focused our investigation on tubulin, the constituent protein of microtubules, as it is a direct target of anesthetics, has explicit relevance to POCD in cytoskeleton-compromised neurodegenerative disorders, and may be the site of a common, unitary mechanism of anesthesia.

Our results indicate that for anesthetics there is a very strong correlation between their potency and the shifts they induce in the characteristic dipole collective modes in the tubulin dimer. Specifically we found tubulin aromatic amino acids alone to possess collective oscillations in networks of London-force dipoles among pi electron resonance clouds of aromatic amino acids in the range of 480 to 700 THz. The presence of an anesthetic (represented as an additional polarizable molecule in the aromatic network) creates another normal mode of the dipolar oscillations. The introduction of an anesthetic also shifts the normal oscillatory modes of the dipoles downward (slower) by an order of 1 to 100 GHz. The most prominent shift observed for the anesthetics is situated around a specific normal mode of oscillation at (613 ± 8) THz. These new oscillatory modes introduced by anesthetics also highly correlate with their anesthetic potency. Interestingly, non-anesthetics follow this trend with a predicted MAC of well over 100% atmospheres, suggesting that this mechanism correctly distinguishes anesthetic and non-anesthetic agents, a requirement for a common anesthetic mechanism. As the anesthetic/convulsant gas flurothyl also lies on this trend line with a clinically relevant MAC, it must be noted that flurothyl can indeed produce anesthesia in mice at a MAC consistent with the Meyer-Overton correlation^27^. The convulsions from flurothyl appear at clinical concentrations an order of magnitude less than the MAC to cause loss of consciousness suggesting an alternate mode and/or target of action for its convulsant properties^27^.

Thus, alterations of the existing oscillatory modes in proteins by anesthetics may be a common mechanism of action, which may tend to alter microtubule polymerization and function. The organizing behavior of macromolecular entities, such as tubulin, indicates the existence of a complex and very fast information processing activity^52^. Indeed, modern experiments suggest that proteins are able to find their cognate partners at a rate 10–100 times faster than allowed by the stochastic dynamics of Brownian motion^53, 54^. One alternative to these purely stochastic interactions is molecules that interact at long range by communicating signals. The assembly of complex macromolecular biological systems from simpler building blocks, as in the case of microtubules, is often driven by weak non-covalent van der Waals or dispersion interactions that arise from electrodynamic correlations between instantaneous charge fluctuations in matter^51, 55–58^.

Indeed, collective excitations and dispersion effects within other macromolecules of biological relevance have been shown by recent experiments^59–61^. These spectral features are commonly attributed to coherent oscillation modes of the whole biomolecule or of a substantial fraction of its atoms. Computational studies of collective electronic motions include normal modes^62^, quasi-harmonic modes^61, 63^, and coarse-grain modes^64^. Comparisons between theory and experiment have yielded consistent results^51, 61^ favoring the presence of such collective motion. These collective conformational vibrations, which are observed in the frequency range of 0.1–1000 Terahertz (THz), bring about oscillations of configurations of electric dipole moments with the calculated timescales for these correlated motions ranging from picoseconds to nanoseconds and beyond^63, 65^.

The collective behavior of the dipole network is a degree of freedom not accounted for by the modeling of simple Coulomb charges. The mode of interest, 613 THz, corresponds to ~2.5 eV, in the visible blue range (489 nm), which is an order of magnitude stronger than typical binding interaction energies (~0.2 eV, about 10 times thermal energy, or kT). These collective dipole oscillations are a global effect due to synchronous/coherent electronic behaviors in tubulin, and such oscillations are energetically relevant by at least two orders of magnitude beyond thermal noise. Theoretically, these long-range dipolar interactions between macromolecules are effective when the interacting system is out of thermal equilibrium^66^, as in most all biological cases. In such a system at physiological temperature two molecules whose dipole moments are “on-resonance” oscillate at the same frequency and undergo an attractive interaction that scales as r^−3^, where r is the distance between molecules^67^. In the “off-resonance” situation, such an attractive interaction produces a standard van der Waals-like potential scaling as r^−6^. Such a frequency-selective interaction, when applied in a biological context such as tubulin self organization and polymerization, is of relevance during the approach of a molecule toward its interacting partner. Shifts in the resonance patterns between tubulin dimers, such as by the addition of an anesthetic molecule as shown here, could “mask” this long range recognition required for ideal polymerization rates by substantially changing the distance dependence from a proportionality of r^−3^ to r^−6^.

Thus, the effect of anesthetic polarizability upon binding in protein hydrophobic pockets has real implications for the general dynamics of protein-protein interactions. While complete depolymerization of microtubules is not necessary for anesthesia, the presence of anesthetic molecules can serve to weaken the integrity of the microtubule structure leading to decreased polymerization rates, thus affecting cytoskeletal reordering in learning, memory, cognition, and long-term potentiation.

In regards to anesthetic action, high-frequency neural oscillations have been associated with conscious states, while low-frequency activity has been associated with unconscious states^68^. Coherence theories of anesthesia suggest that general anesthetics act by disrupting coherent neuronal activity in critical brain structures^69^. On a finer scale, mechanics of individual microtubules contribute to neuronal shape and structure and the physical processes underlying axonal growth cone and dendritic spine motility, as well as intracellular transport. Since microtubule stiffness variation can affect whole cell morphology^70^ and intracellular transport, this could lead to changes in the timing of neuron firing and neuron function, resulting in a loss of coherence and ultimately anesthesia. Controversial theories have been suggested that relate such microtubule processes directly to neural coherence and consciousness^71–73^, but experimental confirmation is needed for validation of such claims. To date, understanding London dispersion and van der Waals interactions in complex systems relies mostly on theoretical concepts and the analysis of the results of computer modeling^74^. While state-of-the-art experiments have provided strong evidence for the many-body nature of dispersion interactions in material surfaces and thin films, it is expected that these effects only “scratch the surface” of the myriad of molecules and materials^74^. Compared to idealized material surfaces and thin films, biomolecules are far more complex and present a significant challenge, as they are heterogeneous in nature and naturally reside in an aqueous environment. As such it would be very challenging and beyond current capabilities to make precise enough measurements to detect polarizability changes as a result of anesthetic binding to any protein.

Thus, with these observations in mind, and in the absence of other suitable single unitary mechanisms of action, we conclude based on our results that anesthesia may be due to alteration of the dipolar oscillations of the electronic degrees of freedom in aromatic molecules in proteins. Anesthetics have the ability to affect these collective dipole oscillations and shift normal mode frequencies due to both their permanent dipoles and electrical polarizabilities. We have examined the effect of anesthetics on the microtubule cytoskeleton due to its potential role in POCD, and its potential to be a common unitary site of anesthetic action. Other proteins do exhibit collective dipole modes in the THz regime, but the energies for the collective oscillations cover a different frequency range owing to difference in the number and arrangement of polarizable amino acids (i.e., for the DNA interacting enzymes EcoR1, and Taq polymerase^57^). Thus, further tests on other candidate sites of anesthetic action (e.g. GABAa, acetylcholine, serotonin, glutamate, glycine, α2-adrenergic, acetylcholine, or adenosine receptors) are warranted to determine if this anesthetic effect is universal or unique to tubulin. While it remains to be seen if this mechanism is general to all proteins, we have shown that this effect may take place in the aromatic networks forming part of the structure of protein subcomponents of neuronal microtubules. Our proposed mechanism may lead to the design and development of novel anesthetic molecules with greater potencies, faster clearance rates, and other desirable properties, while remaining free of potentially detrimental side effects.

## Methods

### Anesthetic Binding Site Prediction

In previous work Craddock *et al*.^32^ predicted putative binding sites for volatile anesthetics on and between tubulin subunits in a microtubule B-type lattice using a combination of homology modeling, molecular dynamics simulations and surface geometry techniques. The homology model was constructed according to Carpenter *et al*.^75^ from the 1JFF^76^ and 1SA0^77^ tubulin structures from the Protein Data Bank^78^ (www.rcsb.org) and simulated according to Craddock *et al*.^32^. The nine most persistent and probable sites from the modeling predictions of Craddock *et al*.^32^ were used in the current study. The largest cluster conformation of the tubulin dimer from the molecular dynamics simulations of Craddock *et al*.^32^ was used for all further calculations in this study.

### Anesthetic Docking to Tubulin

Docking was performed with Molecular Operating Environment (2015.10)^79^ using the Alpha PMI placement method as it is fast and most suited to tight pockets. Each ligand was docked separately in the regions around the nine most persistent and probable anesthetic-binding sites predicted by Craddock *et al*.^32^. The ligand structures were then further refined using the fixed receptor option. This refinement step is an energy minimization using the conventional Amber10:Extended Huckel Theory (EHT) molecular mechanics force field to take electronic effects into account. Partial charges were calculated and reassigned accordingly. During this stage, solvation effects were also calculated using the reaction field functional form for the electrostatic energy term with a dielectric constant equal to 4. The generalized Born solvation model (GB/VI) was used at the end of the refinement step to estimate the final energy. The best conformations were selected using the MOE’s London ΔG scoring function to provide an estimate of the free energy of binding based on the average gain/loss of rotational and translational entropy, the flexibility of the ligand and information about hydrogen bonds. The conformation of each of the anesthetic and non-anesthetic molecules with the best score in each of the nine binding regions was kept for further analysis such that nine final conformations were obtained for each ligand.

### Quantum Chemical Estimate of Molecular Polarizability Tensors

Quantum chemical estimates of the molecular polarizability tensors were obtained for each anesthetic and non-anesthetic molecule in isolation using the best conformation found in each of the nine binding pockets as described above. To calculate the polarizability tensors of the selected anesthetic and non-anesthetic molecules the density functional theory (DFT) method was used with the long-range corrected functional CAM-B3LYP^80^. This method was chosen as it has been found to be very effective in predicting reliable values of polarizability^81^. Calculations were performed with the PolX basis set designed by Sadlej *et al*. for accurate predictions of molecular electric properties including dipole moments and polarizabilities^82–84^. The basis sets were downloaded from http://www.qch.fns.uniba.sk/Baslib/POL. GAMESS-US^85, 86^ was used for all calculations using the finite electric field perturbation method^87, 88^ with default settings in the $FFCALC data group. For the DFT calculations the Janssen’s grid JANS = 2 was used, which generates about 71,000 grid points per atom.

### Calculation of Collective Electronic Behavior

Networks of aromatic amino acids and polarizable molecular agents were modeled as a set of interacting London-force dipoles. Collective dipole oscillations between aromatic amino acids and anesthetic molecules were calculated by considering oriented dipole-dipole interactions between constituent aromatics in the reference frame of tubulin-anesthetic complexes. Following Kurian *et al*.^57, 58^, the polarizability tensors of indole^89^, phenol^90^, and benzene^91–93^ were used to represent the polarizability tensors of the amino acids tryptophan, tyrosine, and phenylalanine, respectively. Polarizability tensors for the anesthetics, non-anesthetics and convulsants were determined as described above. The electronic angular frequencies of induced dipole oscillations were determined as in^57, 58^ from the fundamental dipole relation:

1 $$\overrightarrow{\mu }=\overleftrightarrow{\alpha }\cdot \overrightarrow{E}$$

Here, we only consider the diagonal elements of the polarizability tensors ***α***_*xx*_, ***α***_*yy*_, ***α***_*zz*_, and neglect off- diagonal terms. After alignment of the polarizabilities with the orientation of the aromatic amino acids in the protein coordinate space, we take the magnitude of the mean polarizability to be $\bar{\alpha }=\frac{1}{3}({\alpha }_{xx}+{\alpha }_{yy}+{\alpha }_{zz})$ where the induced dipole direction for each aromatic is defined by the vector $\vec{\alpha ^{\prime} }=({\alpha }_{xx}^{^{\prime} },{\alpha }_{yy}^{^{\prime} },{\alpha }_{zz}^{^{\prime} })$ in the protein coordinate frame.

The average angular frequency for the dipole oscillation is determined from $\bar{\omega }=\sqrt{{\omega }_{xx}^{2}+{\omega }_{yy}^{2}+{\omega }_{zz}^{2}}$, where the elements of the angular frequency tensor for each amino-acid dipole are determined from polarizability data using:

2 $${\omega }_{ii}=\sqrt{\frac{{e}^{2}}{{m}_{e}{\alpha }_{ii}^{^{\prime} }}}$$

where the mass *m*_*e*_ and charge *e* of an electron are used to approximate the charge-separated dipoles of the amino acids.

Considering the collection of aromatics to be a network of *N* harmonic oscillators coupled via induced dipole interactions, the resulting Hamiltonian for such a collection of *N* aromatic amino acid induced dipoles is then:

3 $$\begin{array}{ccc}H & = & T+V\\  & = & \sum _{n=0}^{N-1}(\frac{{p}_{n}^{2}}{2{m}_{e}}+\frac{{m}_{e}{\bar{\omega }}_{n}^{2}}{2}{d}_{n}^{2})+\frac{1}{2}\sum _{n\ne m\,n,m=0}^{N-1}\frac{1}{4\pi {\varepsilon }_{0}}(\frac{{\vec{\mu }}_{n}\cdot {\vec{\mu }}_{m}-3({\vec{\mu }}_{n}\cdot {\hat{r}}_{nm})({\vec{\mu }}_{m}\cdot {\hat{r}}_{nm})}{|{r}_{nm}^{3}|})\end{array}$$

where the first term describes the kinetic energy of the system, the second term describes the harmonic oscillator potentials with displacement coordinates ${\vec{d}}_{n}=({x}_{n},{y}_{n},{z}_{n})$ defined between each delocalized electron cloud and its amino acid core, and the third term describes the pairwise interactions between each amino acid induced dipole in the tubulin aromatic network.

Following^57, 58^ the collective eigenmode frequencies for the oscillations are obtained from the symmetric longitudinal potential matrix *V* and the diagonal kinetic matrix *T* for the aromatic network. The problem then consists of solving the characteristic equation for matrix eigenvalues:

4 $$det(V-{\Omega }_{n}^{2}T)=0$$

Using numerical packages in Python, the eigenvalues of *V* were solved. As *T* is diagonal, these eigenvalues correspond to ${m}_{e}{\Omega }_{n}^{2}$.

### Data availability

The datasets generated during and/or analyzed during the current study are available from the corresponding author on reasonable request.

## Electronic supplementary material

Supplementary Information
