# Supplementary material for: Anesthetic Alterations of Collective Terahertz Oscillations in Tubulin Correlate with Clinical Potency: Implications for Anesthetic Action and Post-Operative Cognitive Dysfunction
Source: Sci Rep. 2017 Aug 29;7:9877. doi: 10.1038/s41598-017-09992-7 (PMC5575257; doi:10.1038/s41598-017-09992-7)
Supplement: Supplementary file 1 — Supplementary Information [file 41598_2017_9992_MOESM1_ESM.doc]

**Anesthetic Induced Alterations in London Dispersion Interactions in Tubulin Correlate with Their Potency Implying a Novel Mode of Action**

Travis J. A. Craddock, Philip Kurian, Jordane Preto, Kamlesh Sahu, Stuart R. Hameroff, Mariusz Klobukowski, and Jack A. Tuszynski

**SUPPLEMENTARY INFORMATION**

**Table S1: MAC, Oil:Gas Partition Coefficient (λ), and calculated mean polarizability ($\bar{\alpha}$) for select anesthetics, non-anesthetics and convulsants. *MAC for non-anesthetics and convulsants are predicted from the Meyer-Overton correlation.**

| **Molecule** | **MAC (%)** | **λ (Oil:Gas)** | $\bar{\alpha}$ **(Å^3^)** |
| --- | --- | --- | --- |
| ***Anesthetics*** | | | |
| Desflurane | 6.00 | 19 | 7.22 |
| Diethylether | 1.90 | 65 | 8.45 |
| Enflurane | 1.70 | 98 | 9.04 |
| Halothane | 0.77 | 224 | 9.38 |
| Isoflurane | 1.20 | 96 | 9.10 |
| Methoxyflurane | 0.16 | 960 | 10.70 |
| Nitrous Oxide | 101.00 | 0.39 | 2.86 |
| Sevoflurane | 2.00 | 53 | 8.95 |
| ***Non-anesthetics*** | | | |
| F6 | 3.00^*^ | 44 | 11.50 |
| Trifluromethylbenzene | 0.80^*^ | 264 | 16.37 |
| ***Convulsants*** | | | |
| Flurothyl | 2.50^*^ | 47 | 8.83 |

**Table S2: MOE binding scores for select anesthetics, non-anesthetics and convulsants to predicted sites on tubulin. ^*^Numbering scheme according to ^31^**.

|  | | **MOE Binding Scores (kJ/mol)** | | | | | | | | |
| --- | --- | --- | --- | --- | --- | --- | --- | --- | --- | --- |
| **Tubulin Binding Site^*^** | **1** | | **4** | **5** | **7** | **21** | **23** | **37** | **38** | **39** |
| ***Anesthetics*** | | | | | | | | | | |
| Desflurane | -7.83 | | -7.94 | -9.16 | -8.96 | -5.94 | -7.29 | -6.69 | -7.31 | -7.00 |
| Diethylether | -5.87 | | -7.30 | -7.15 | -7.20 | -5.02 | -5.70 | -5.58 | -7.55 | -5.29 |
| Enflurane | -7.71 | | -8.24 | -8.73 | -8.48 | -6.33 | -7.19 | -7.38 | -9.19 | -7.40 |
| Halothane | -7.48 | | -6.29 | -6.72 | -7.91 | -5.30 | -6.36 | -6.36 | -7.40 | -6.28 |
| Isoflurane | -7.67 | | -8.28 | -8.46 | -8.90 | -5.97 | -7.41 | -7.19 | -8.92 | -7.35 |
| Methoxyflurane | -7.25 | | -8.24 | -8.18 | -7.84 | -5.80 | -6.52 | -6.42 | -6.82 | -6.79 |
| Nitrous Oxide | -4.55 | | -4.41 | -6.16 | -5.46 | -4.95 | -4.97 | -4.97 | -6.07 | -4.43 |
| Sevoflurane | -8.51 | | -9.10 | -7.46 | -8.53 | -6.23 | -7.87 | -7.61 | -8.36 | -7.60 |
| ***Non-anesthetics*** | | | | | | | | | | |
| F6 | -7.88 | | -7.73 | NaN | -8.60 | -6.12 | -6.59 | -7.83 | -8.37 | -7.88 |
| Trifluromethylbenzene | -10.72 | | -9.32 | -8.32 | NaN | -6.82 | -8.99 | -9.36 | -11.06 | -9.37 |
| ***Convulsants*** | | | | | | | | | | |
| Flurothyl | -8.22 | | -9.74 | -9.64 | -9.51 | -6.17 | -7.49 | -7.70 | -7.89 | -7.44 |

**
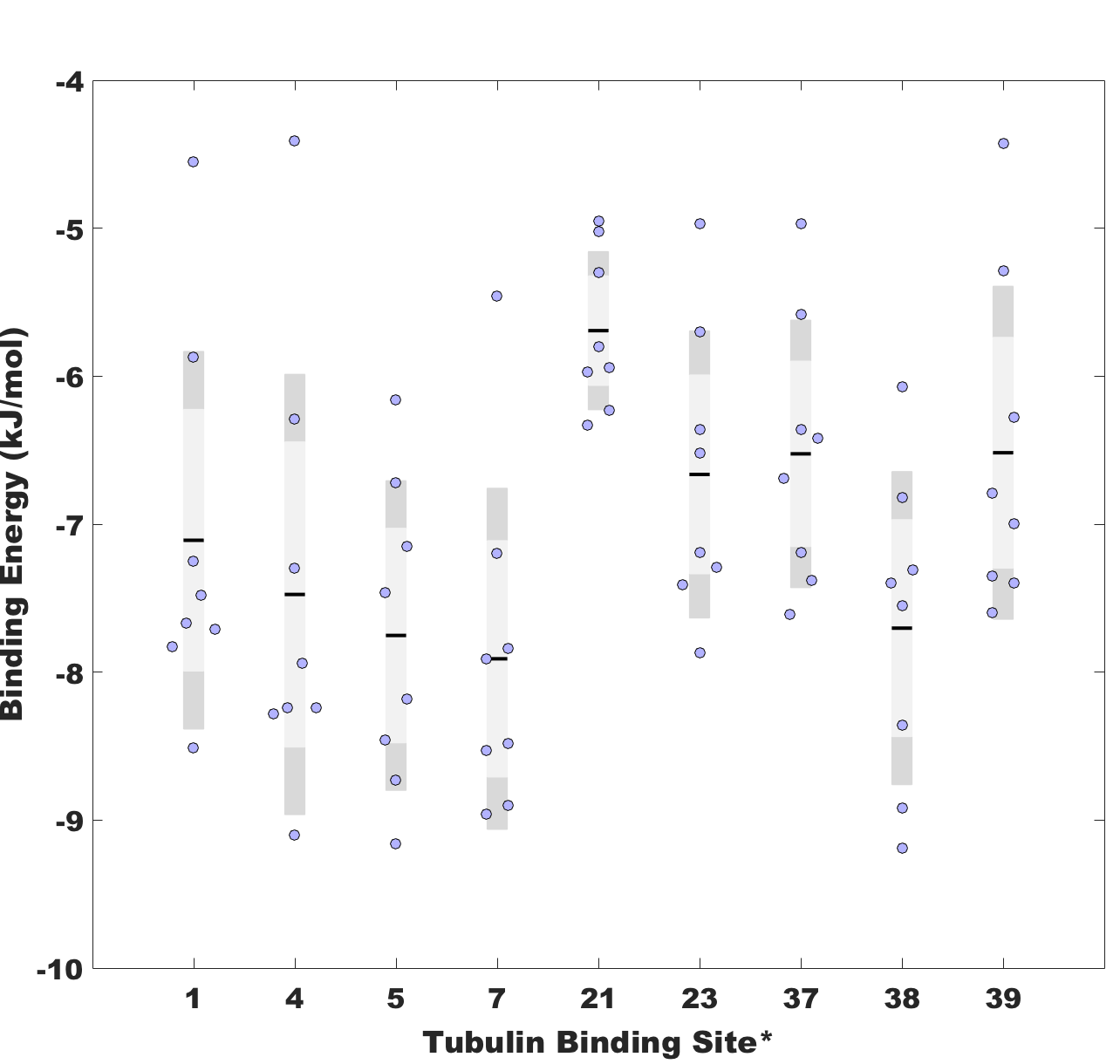
**

**Figure S1:** **Docking results of ligands to tubulin.** Scatter plots showing predicted binding scores (kJ/mol) for anesthetics to 9 predicted binding sites on tubulin. More negative MOE score indicates stronger binding. The black line represents the mean, while the light gray box covers the standard error of the mean, and the dark gray box covers one standard deviation of the data.
